# The Inhibitory Effect of Quercetin on Asymmetric Dimethylarginine-Induced Apoptosis Is Mediated by the Endoplasmic Reticulum Stress Pathway in Glomerular Endothelial Cells

**DOI:** 10.3390/ijms15010484

**Published:** 2014-01-02

**Authors:** Weikang Guo, Jiaxiang Ding, Aihua Zhang, Wendi Dai, Sha Liu, Zongli Diao, Liyan Wang, Xue Han, Wenhu Liu

**Affiliations:** Department of Nephrology, Beijing Friendship Hospital, Faculty of Kidney Diseases, Capital Medical University, Beijing 100050, China; E-Mails: gwk0777@gmail.com (W.G.); mattding@163.com (J.D.); zhah0351@126.com (A.Z.); dianadwd@163.com (W.D.); liusha201304@163.com (S.L.); diaoted@163.com (Z.D.); wangliyan7731@sina.com (L.W.); whysnowman@126.com (X.H.)

**Keywords:** ER stress, apoptosis, ADMA, TGF-β, quercetin

## Abstract

Asymmetric dimethylarginine (ADMA) is considered an independent mortality and cardiovascular risk factor in chronic kidney disease (CKD) patients, and contributes to the development of renal fibrosis. Quercetin (QC), a natural component of foods, protects against renal injury. Here, we explored the possible mechanisms that are responsible for ADMA-induced renal fibrosis and the protective effect of QC. We found that ADMA treatment activated the endoplasmic reticulum (ER) stress sensor proteins phosphorylated protein kinase RNA-activated-like ER kinase (PERK) and inositol requiring-1α (IRE1), which correspondingly induced C/EBP homologous protein (CHOP) expression and phosphorylated c-Jun *N*-terminal kinase (JNK) phosphorylation in glomerular endothelial cells (GEnCs). Following this, ADMA promoted ER stress-induced apoptosis and resulted in transforming growth factor β (TGF-β) expression in GEnCs. SP600125, an inhibitor of JNK, and CHOP siRNA protected against ADMA-induced cell apoptosis and TGF-β expression. QC prevented ADMA-induced PERK and IRE1 apoptotic ER stress pathway activation. Also, ADMA-induced GEnCs apoptosis and TGF-β expression was reduced by QC. Overexpression of CHOP blocked QC-mediated protection from apoptosis in ER stressed cells. Overall, these observations indicate that ADMA may induce GEnCs apoptosis and TGF-β expression by targeting the PERK-CHOP and IRE1-JNK pathway. In addition, drugs such as QC targeting ER stress may hold great promise for the development of novel therapies against ADMA-induced renal fibrosis.

## 1. Introduction

Asymmetric dimethylarginine (ω-N^G^, N^G^-asymmetric dimethylarginine; ADMA) is a residue of the proteolysis of arginine methylated proteins, and an endogenous competitive inhibitor of nitric oxide synthase (NOS) enzymes at supraphysiological concentrations, although the physiological relevance of this inhibition remains unclear [1]. Many studies have reported on the accumulation of ADMA in chronic kidney disease (CKD) patients. Indeed, blood concentrations of ADMA are markedly increased at a very early stage, even when glomerular filtration rate (GFR) is still within the normal range [2]. Increased ADMA induces glomerular and tubulointerstitial fibrosis and accelerates renal dysfunction progression [3–6].

Apoptosis of glomerular endothelial cells (GEnC) is known to correlate directly with the development of glomerular scarring, which eventually leads to glomerular sclerosis [7,8]. However, the mechanism of GEnC apoptosis is not well understood. Evidence suggests that endoplasmic reticulum (ER) stress plays an important role in the decision of cell survival and death [9]. ER stress is triggered when unfolded or misfolded proteins are overloaded in the ER. To improve and maintain ER function against such stress, cells adapt themselves via the unfolded protein response (UPR), such as attenuation of general translation, induction of ER chaperones and foldases, and activation of ER-associated degradation (ERAD) to eliminate immature proteins. However, when ER stress is either severe or prolonged, apoptosis programs are activated to remove damaged cells. One of the mechanisms of ER stress-induced cell death involves protein kinase RNA-activated-like ER kinase (PERK)-mediated eukaryotic translation initiation factor 2α (eIF2α) phosphorylation, preferential translation of activating transcription factor 4 (ATF4) and induction of C/EBP homologous protein (CHOP, also called growth arrest and DNA-damage-inducible 153, GADD153). Another apoptotic ER stress pathway is triggered by inositol requiring-1α (IRE1), which activates apoptosis signal-regulating kinase-1 and c-Jun *N*-terminal kinase (JNK), eventually inducing cell apoptosis [9,10]. Until now, it is still unknown whether ER stress participates in GEnC apoptosis to accelerate the progression of renal fibrosis. Therefore, in this study we investigated the possible involvement of the ER stress signaling pathway in ADMA-induced apoptosis in GEnCs.

Quercetin (QC), one of the most important flavonoids, has been extensively studied because of its wide distribution in various foods. In addition to its antioxidant effect, there are an impressive number of enzymes whose activities are modulated (mostly inhibited) by QC [11]. The modulation of certain cell functions by QC may contribute to its beneficial effects. Evidence from *in vitro* and animal studies indicated that QC exerts a broad range of pharmacological effects, such as anti-platelet aggregation, endothelium-independent vasodilator effects, anti-hypertension, lipoxygenase inhibition and reduction of adhesion molecules and other inflammatory markers [11]. Especially in recent years, many reports have indicated that QC showed protective effects on renal injury [12–16]. To date, most research has focused on QC’s antioxidant effects; however, QC can potentially modulate many molecules. Thus, it may act by multiple mechanisms in the protection of cells from injury. The precise mechanisms by which QC exerts its positive effects on renal disease have yet to be elucidated. Therefore, we explored the protective mechanism of QC on ADMA-induced GEnC apoptosis. In particular, we characterized: (i) ADMA induced cell apoptosis and TGF-β expression in GEnCs; (ii) ER stress pathways participated in ADMA-induced GEnC apoptosis; and (iii) QC inhibited of ADMA-induced GEnC apoptosis and TGF-β expression by targeting ER stress pathways.

## 2. Results

### 2.1. QC Inhibited ADMA-Induced Apoptosis in GEnCs

**Figure 1.**
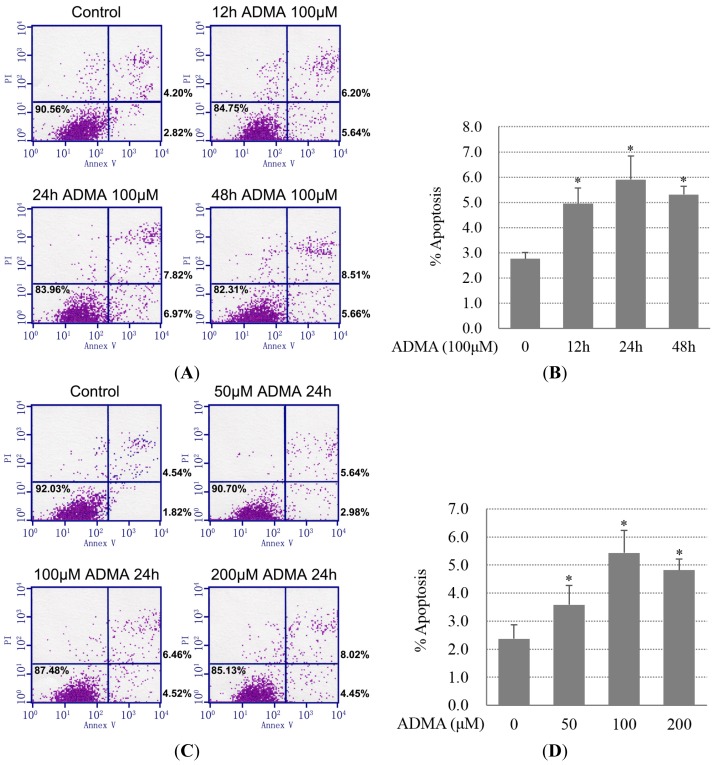

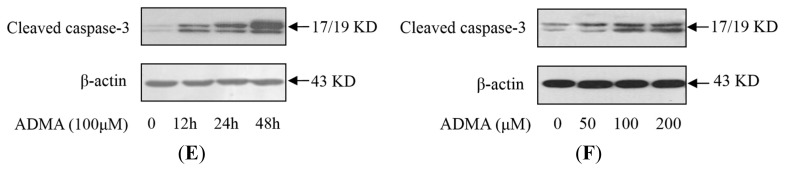
Asymmetric dimethylarginine (ADMA) induced glomerular endothelial cells (GEnC) apoptosis. (**A**,**B**) Primary GEnCs were incubated with 100 μM ADMA for 12, 24 or 48 h. Annexin V-FITC and PI reagent were added and 10,000 cells were analyzed on a fluorescence-activated cell sorting (FACS) Calibur instrument. Percentages of annexin V-positive/PI-negative or annexin V-positive/PI-positive cells are shown; (**C**,**D**) Similar analysis performed in GEnCs treated with 50, 100 or 200 μM ADMA for 24 h. In (**A**) and (**C**), representative results out of three are shown; (**B**,**D**) data showed the mean SD of three independent experiments. *p* < 0.05 by ANOVA was considered significant. * different from controls (*p* < 0.05); and (**E**,**F**) Cells were treated with ADMA at the indicated dose (0–200 μM) and time period (0–48 h). Cell extracts were subjected to western blotting using antibody against cleaved caspase-3.

Apoptotic (annexin V^+^/PI^−^), dead (annexin V^+^/PI^+^) and viable cells (annexin V^−^/PI^−^) were separated on the basis of double labeling for annexin V-FITC and PI, a membrane DNA stain. It was revealed that treatment with ADMA (100 μM) for 12–48 h increased the percentage of apoptotic cells (Figure 1A,B). Cells treatment with 24 h showed the maximum number of apoptotic cells. However, the percentage of dead cells (annexin V^+^/PI^+^) increased in a time-dependent manner (Figure 1A). Moreover, in comparison with different doses of ADMA (50–200 μM)-treated cells for 24 h, 100 μM ADMA-treated GEnCs displayed the highest percentage of apoptotic cells (Figure 1C,D).

Further, we analyzed the cleaved caspase-3, a critical executioner of apoptosis. We found that ADMA increased cleaved caspase-3 expression in time- and dose-dependent manner (Figure 1E,F).

### 2.2. Role of ER Stress in ADMA-Mediated GEnC Apoptosis

**Figure 2.**
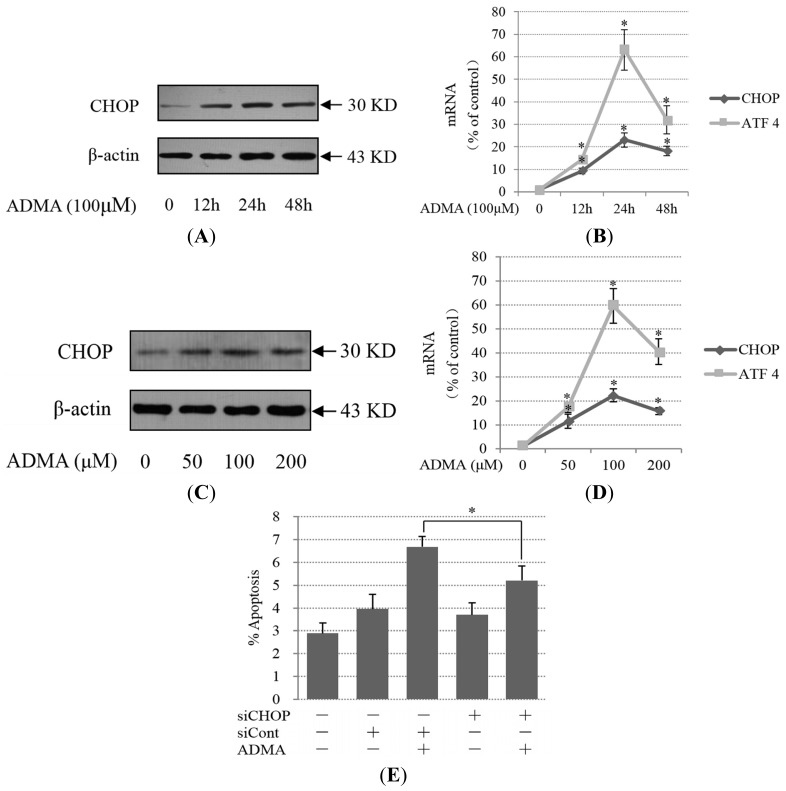
Role of PERK-CHOP ER stress pathway in ADMA-induced GEnC apoptosis. Cells were treated with ADMA at the indicated dose (0–200 μM) and time period (0–48 h). To analyze CHOP expression, cell extracts or RNA were subjected to western blotting (**A**,**C**) or RT-PCR (**B**,**D**); and (**E**) After transfection with siRNA, GEnCs were exposed to 100 μM ADMA for 24 h. Apoptosis was assessed by flow cytometry after annexin V and PI staining (mean ± SD, *n* = 3). *p* < 0.05 by ANOVA was considered significant. * different from controls (*p* < 0.05).

The ER stress pathway was recently shown to be directly involved in the induction of cell apoptosis, and previous studies have identified the induction of ER stress in models of membranous nephropathy [17], nephrotic syndrome [18], and focal segmental glomerulosclerosis [19]. Therefore, we examined whether ER stress was involved in ADMA-mediated GEnC apoptosis. One component of the proapoptotic signal generated by ER stress is delivered via PERK, with downstream activation of ATF4 and CHOP. We found that the expression of ATF4 mRNA, CHOP protein and mRNA were increased after treatment with 100 μM ADMA for 12–48 h (Figure 2A,B). The induction of CHOP and ATF4 reached a peak after treatment with ADMA for 24 h (Figure 2A,B). Furthermore, even when GEnCs were exposed to a relatively low dose of ADMA (50 μM) for 24 h, CHOP and ATF4 expression was higher than that of the control groups (Figure 2C,D). The time-curve and dose-curve results of CHOP and ATF4 expression are generally consistent with prior results of the apoptosis analysis. To assess the role of CHOP as a potential mediator of GEnC apoptosis, GEnCs were transfected with siRNA for CHOP (siCHOP) or control siRNA (siCont). siCHOP transfection decreased CHOP expression in GEnCs exposed to ADMA when compared with control siRNA-transfected cells (Supplementary Figure S1). Reduced CHOP expression by siCHOP resulted in protection against ADMA-induced apoptosis (Figure 2E).

**Figure 3.**
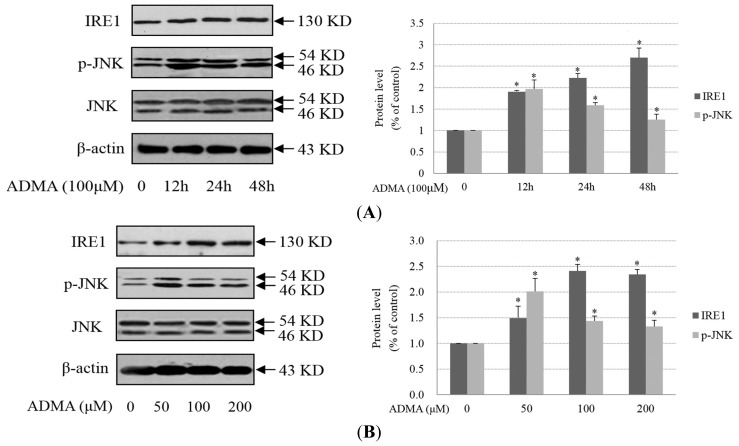

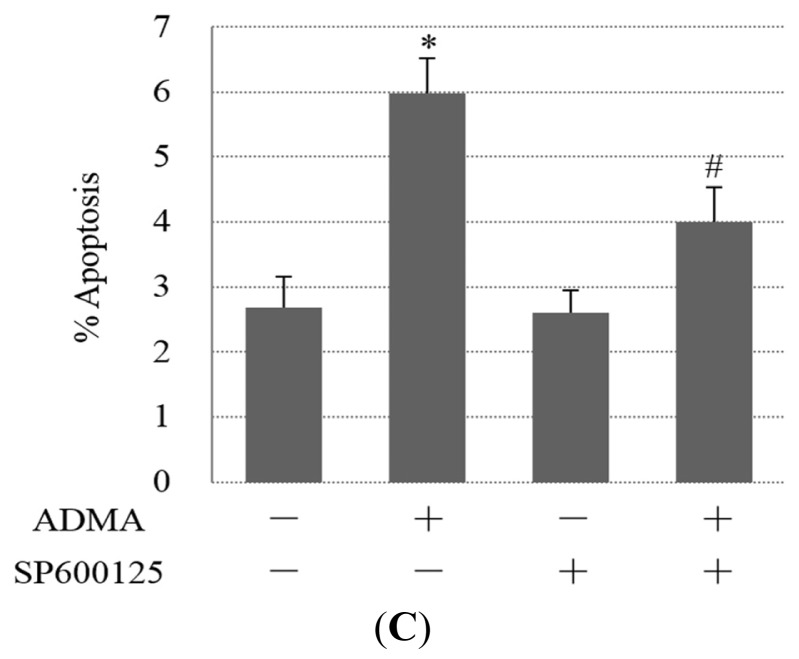
Role of IRE1-JNK ER stress pathway in ADMA-induced GEnC apoptosis. (**A**,**B**) Cells were treated with ADMA at the indicated dose (0–200 μM) and time period (0–48 h). Cells extracts were subjected to western blotting using antibodies against IRE1 and p-JNK. Results are the mean ± SD (*n* = 3); (**C**) GEnC was pretreated with 10 μM SP600125 for 30 min and then exposed to 100 μM ADMA for 24 h. Apoptosis was assessed by flow cytometry after annexin V and PI staining (mean ± SD, *n* = 3). *p* < 0.05 by ANOVA was considered significant. * different from controls (*p* < 0.05); # different from ADMA-stimulated controls (*p* < 0.05).

Prolonged activation of IRE1 can also trigger apoptosis in cells under certain physiologic and pathophysiologic conditions. IRE1 promotes a cascade of phosphorylation events that ultimately activates JNK. Given the links between JNK activity and apoptosis, JNK activity may link IRE1-mediated ER stress signaling to cell death. We found that ADMA increased IRE1 protein expression and JNK phosphorylation levels (Figure 3A,B). The time-curve and dose-curve results of IRE1 expression were basically consistent with previous results of the apoptotic analysis (Figure 1A,B). In contrast with IRE1 expression, JNK phosphorylation reached the plateau at 12 h upon 100 μM ADMA treatment in GEnCs. When GEnCs were exposed to different doses of ADMA (50–200 μM), p-JNK was at its highest level under 50 μM of ADMA treatment (Figure 3A,B). The different time-curve and dose-curve data of JNK phosphorylation compared with IRE1 expression indicated that other factors may also regulate JNK activation in ADMA-induced GEnC apoptosis. SP600125, a specific inhibitor of JNK, protected cells from ADMA-induced apoptosis (Figure 3C). All these findings suggest that both PERK-CHOP and IRE1-JNK ER stress pathways are involved in ADMA-induced apoptosis in GEnCs.

### 2.3. QC Suppressed ADMA-Induced ER Stress in GEnCs

**Figure 4.**
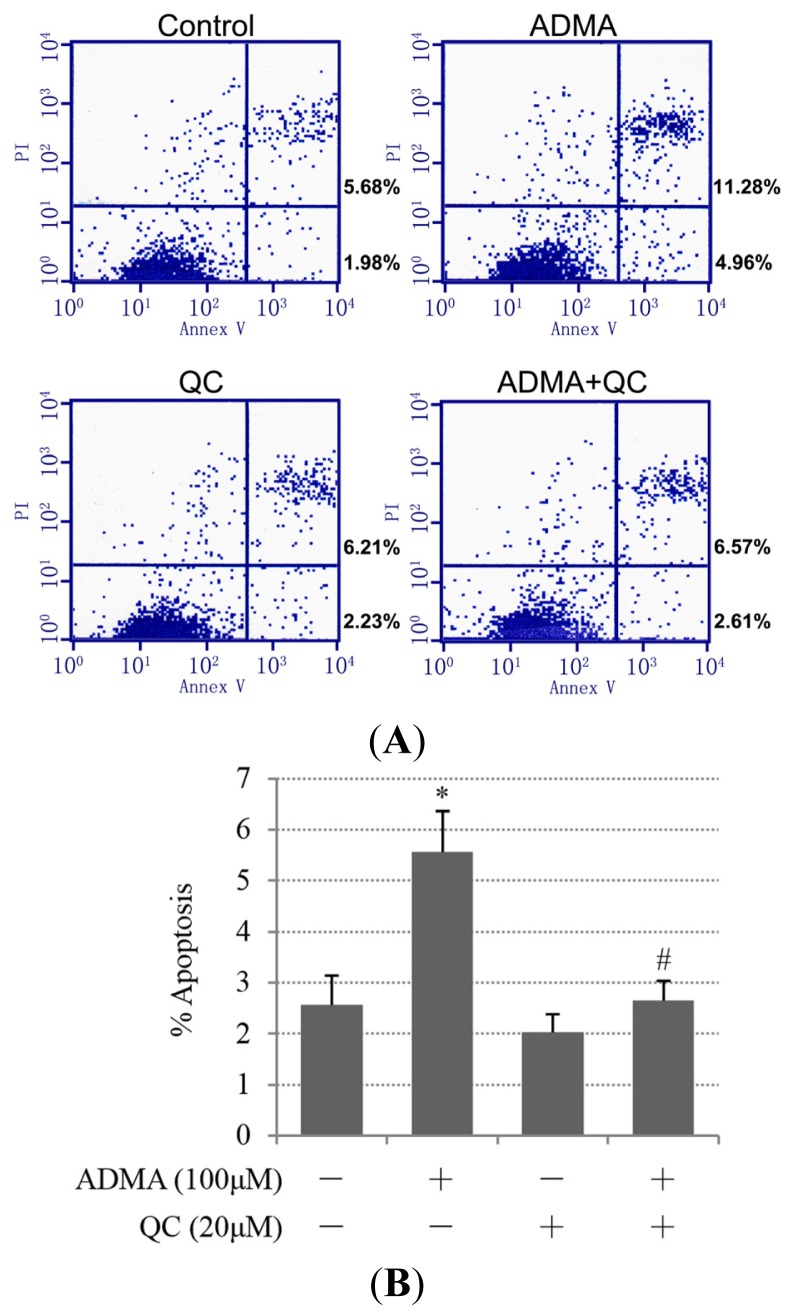
Quercetin (QC) inhibited ADMA-induced apoptosis. GEnCs were treated for 1 h in the presence or absence of 20 μM QC. Cells were then incubated with 100 μM ADMA for 24 h. Apoptosis was assessed by flow cytometry. Data showed one representative experiment (**A**) or the mean SD of three independent experiments (**B**). *p* < 0.05 by ANOVA was considered significant. * different from controls (*p* < 0.05); # different from ADMA-stimulated controls (*p* < 0.05).

**Figure 5.**
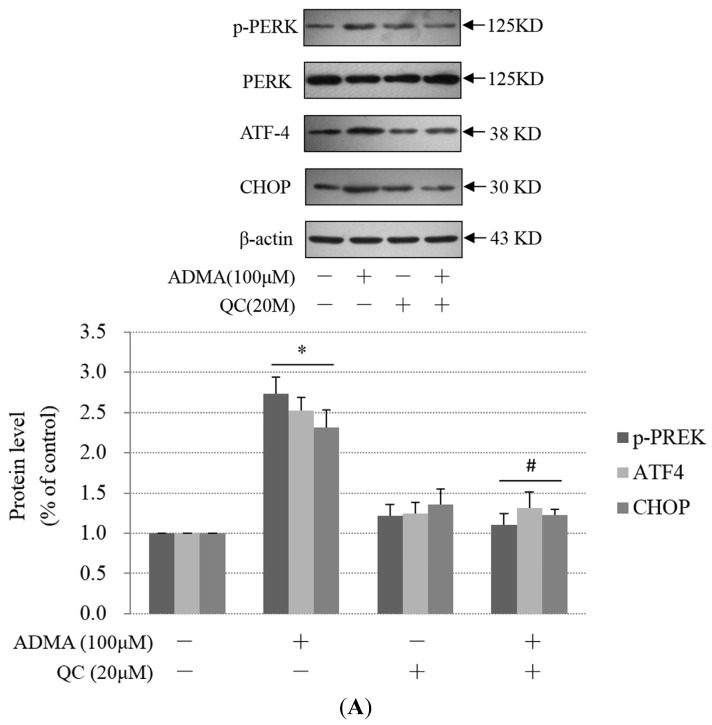

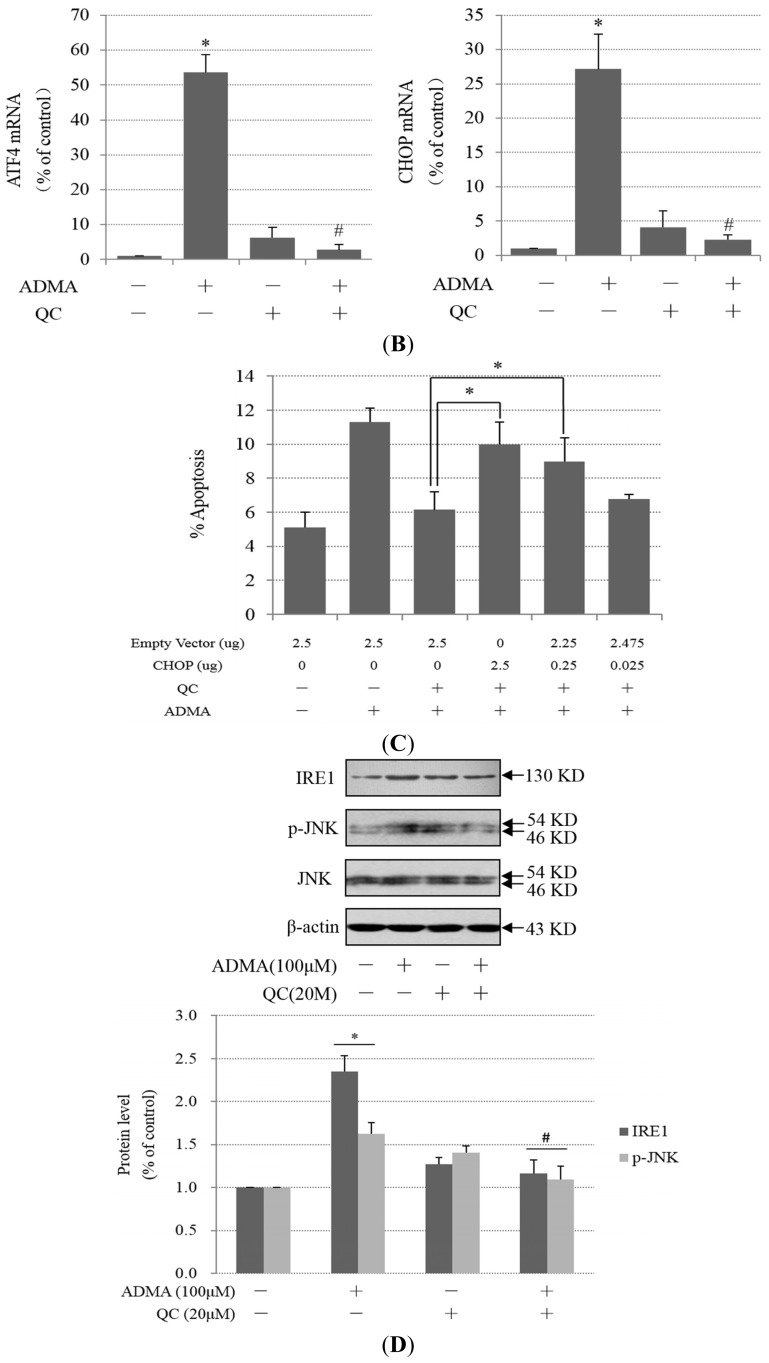

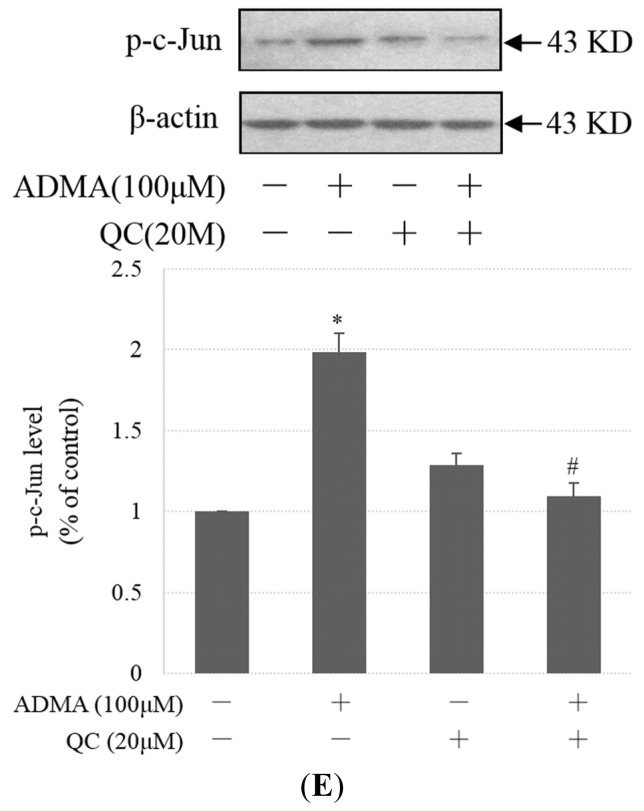
QC inhibited ADMA-induced ER stress. (**A**) QC prevented the ER stress pathway by down-regulating the expression of PERK-ATF4-CHOP. GEnCs were treated for 1 h in the presence or absence of 20 μM QC prior to incubation with 100 μM ADMA for 24 h. Cells were then lysed, and levels of Thr 981 phosphorylated PERK (p-PERK), ATF4, and CHOP were assayed by western blot analysis. β-Actin or PERK is shown as a loading control. Results are the mean ± SD (*n* = 3); (**B**) Real-time PCR analysis of ATF4 and CHOP mRNA levels in cells exposed to ADMA (100 μM) for 24 h in the absence or presence of QC (means ± SD, *n* = 3); (**C**) Cells were transfected with graded concentrations of *pcDNA3-GADD153* expression vectors or the control empty vector, and incubated for 24 h. Cells were pretreated with 20 μM QC for 1 h and then restimulated with 100 μM ADMA for a further 24 h. Apoptosis was assessed by flow cytometry (mean ± SD, *n* = 3); and (**D**,**E**) QC inhibited ADMA-induced IRE1 expression, JNK phosphorylation and c-Jun phosphorylation. GEnC were treated for 1 h in the presence or absence of 20 μM QC before incubation with 100 μM ADMA for 24 h. Cells were then lysed, and levels of IRE1, p-JNK and p-c-Jun were assayed by western blot analysis. Results are the mean ± SD (*n* = 3). *p* < 0.05 by ANOVA was considered significant. * different from controls (*p* < 0.05); # different from ADMA-stimulated controls (*p* < 0.05).

Some studies have suggested that QC has a renal-protective role against kidney disease [12–16]. To test the hypothesis that QC suppresses ADMA-induced GEnC apoptosis and exerts a renal-protective effect, GEnCs were pretreated in the absence or presence of 20 μM QC and then subjected to ADMA stimulation. QC pre-treatment markedly decreased ADMA-induced apoptosis (Figure 4A,B). We speculated that the anti-apoptotic effect of QC on ADMA-treated GEnCs was due to ER stress suppression. Consistent with our speculation, PERK phosphorylation, ATF4 and CHOP protein expression were reduced in the QC-pretreated group when compared with ADMA treatment alone (Figure 5A). Furthermore, the induction of ATF4 and CHOP mRNA by ADMA was also reduced after QC treatment (Figure 5B). We next used the CHOP/GADD153 cDNA-containing plasmid (pcDNA3.1-GADD153) to overexpress CHOP. After transfection with pcDNA3.1-GADD153, CHOP expression increased (Supplementary Figure S2). Transfection with pcDNA3.1-GADD153 restored CHOP expression in QC-pretreated, ADMA-stimulated GEnCs to a level similar to that in GEnCs, which were not pretreated with QC (data not shown). Cell apoptosis was reinstated in cells transiently transfected with pcDNA3.1-GADD153 in a dose-dependent manner (Figure 5C), which indicated that restoration of CHOP prevented QC-mediated protection from ADMA-induced apoptosis. Treatment with pcDNA3.1-GADD153 alone did not lead to an increase of GEnC apoptosis (data not shown). Thus, these data suggest that the suppression of CHOP may be one of the mechanisms by which QC prevents ADMA-induced GEnC apoptosis.

Next, we investigated whether the IRE1 branch of the UPR was also inhibited by QC. We found that QC also inhibited ADMA-induced IRE1 expression and JNK phosphorylation. (Figure 5D). The level of p-c-Jun, a major downstream target of JNK, was also increased after treatment with ADMA. Unsurprisingly, this effect was weakened by QC pretreatment (Figure 5E).

### 2.4. QC Inhibits ADMA-Induced TGF-β Expression in GEnCs

**Figure 6.**
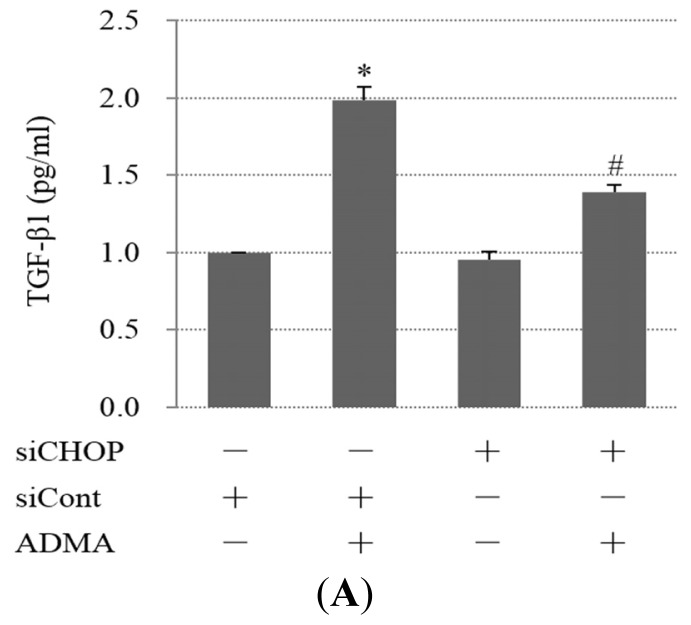

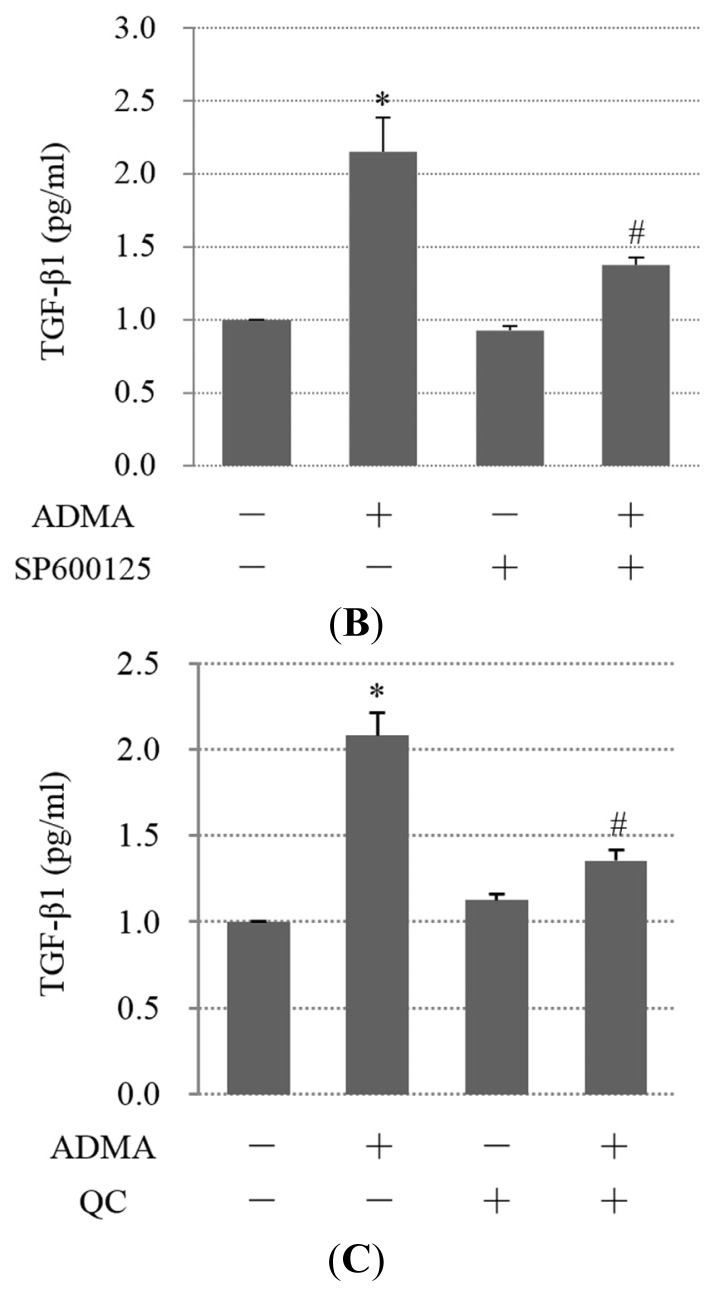
ADMA-induced TGF-β1 expression through ER stress and inhibition by QC treatment. (**A**) GEnCs transfected with siRNA were exposed for 24 h to 100 μM ADMA. TGF-β1 levels were measured by ELISA (mean ± SD, *n* = 3); (**B**) GEnCs were pretreated with 10 μM SP600125 for 30 min and then exposed to 100 μM ADMA for 24 h. TGF-β1 levels were measured by ELISA (mean ± SD, *n* = 3); and (**C**) GEnCs were treated for 1 h in the presence or absence of 20 μM QC and then incubated with 100 μM ADMA for 24 h. Culture supernatants were collected and analyzed for TGF-β1 levels using an ELISA kit. *p* < 0.05 by ANOVA was considered significant. * different from controls (*p* < 0.05); # different from ADMA-stimulated controls (*p* < 0.05).

To investigate whether ER stress is involved in renal fibrosis, the expression of TGF-β was examined in GEnCs. Our results showed that ADMA induced the expression of TGF-β, while down-regulation of CHOP by siRNA or inhibition of JNK by SP600125 decreased the expression of TGF-β. This result indicates that CHOP and JNK are mediators of ADMA-induced TGF-β expression (Figure 6A,B). Furthermore, we found that QC apparently inhibited ADMA-induced TGF-β expression (Figure 6C). Therefore, QC may exert an anti-fibrotic effect by inhibiting TGF-β expression.

## 3. Discussion

Apoptosis of GEnCs is an important factor involved in the development of glomerular sclerosis [7]. Accumulation of ADMA has been shown to promote glomerular sclerosis in a rat model of CKD [20]. However, whether ADMA directly induces GEnC apoptosis *in vitro* is unknown. In this study, we demonstrated that ADMA promoted GEnC from apoptosis. This may be one of the mechanism(s) whereby ADMA promotes the development of glomerular sclerosis.

Apoptosis is a genetically programmed mechanism that allows cells to commit suicide. It has been shown that it can be initiated or inhibited by a variety of environmental stimuli, both physiological and pathological. The extrinsic and intrinsic pathways represent two major well-studied apoptotic processes. The extrinsic pathway, exemplified by the ligation of tumor necrosis factor α receptor (TNFR), leads to self-association of cell-surface receptors and the recruitment of caspases to the activated receptor. By contrast, intrinsic apoptotic stimuli acts chiefly on the Bcl-2 family of proteins. The center of this pathway is cytochrome-c, which is released from mitochondria [21]. However, it is against this backdrop that the initiation of apoptosis by ER stress further clouds the comprehensive understanding of the initiation of apoptosis. The ER is the site of synthesis, folding and modification of secretory and cell-surface proteins. ER dysfunction causes aberrant protein folding in the ER lumen. The accumulation of these aberrant unfolded proteins in turn induces ER stress, which upregulates the capacity of the ER to process abnormal proteins [22,23]. ER stress initially aims to promote cell survival, but if ER stress persists or is prolonged, it also activates pathways leading to cell death.

Recent investigations suggested roles of ER stress in some types of glomerular disorders, especially proteinuric diseases caused by injury of podocytes [23]. But whether ER stress is involved in the apoptosis in GEnCs is unknown. In this study, we first found that the PERK branch of the UPR was involved in ADMA-induced apoptosis in GEnCs. We found that ADMA induced PERK expression and selectively activated the proapoptotic protein CHOP. SiRNA targeting CHOP effectively inhibited ADMA-induced GEnC apoptosis, indicating that the PERK-CHOP pathway plays an important part in ADMA-induced GEnC apoptosis and that drugs targeting PERK-CHOP may alleviate ADMA-induced apoptosis. In addition, we noticed that knocking down CHOP did not decrease cell apoptosis to control levels. Therefore, we examined whether the IRE1 branch of the UPR was also involved in ADMA-induced apoptosis. We found that ADMA induced IRE1 expression and JNK phosphorylation. Our results indicated that two apoptotic ER stress pathways were involved in ADMA-triggered GEnC apoptosis.

In the normal population, plasma levels of ADMA are 0.92–0.11 μM [24,25]. Plasma levels of ADMA increase during pre-eclampsia, hypertension, hypertriglyceridemia, end-stage renal disease, type 2 diabetes, and congestive heart failure. Hemodialysis (HD)-treated patients with atherosclerosis exhibit significantly higher ADMA levels of up to 7.31 μM [26]. After long-term accumulation in the human body, ADMA gives rise to a number of pathophysiological changes. When we performed experiments to study the toxicity mechanisms of ADMA *in vitro*, much larger doses than pathological concentrations in clinical patients were required. Previous studies have shown that 100 μM ADMA is less toxic, but maximizes the biological effect *in vitro* [27–30]. The effects of low doses of ADMA in conjunction with long-term stimulation upon ER stress activation still need to be further studied *in vivo*.

QC is a phytochemical compound belonging to the flavonoid family and is the most ubiquitous of the dietary flavonoids. QC is reported to be a potent antioxidant [31,32] and to inhibit enzyme activity [33], inflammatory processes [34], and adhesion molecule expression [35]. In the clinic, QC is used to treat a variety of diseases such as allergies, asthma, bacterial infections, arthritis, gout, eye disorders, hypertension, neurodegenerative disorders and CKDs. In animal experiments, the administration of QC has been shown to be of benefit in the prevention and attenuation of renal injury in numerous models of kidney disease [13–16]. Treatment with QC significantly attenuated renal dysfunction and oxidative stress in diabetic rats [13], and was protective against ischemia/reperfusion-induced renal injury, cyclosporine (CsA)-induced renal dysfunction and chronic cadmium nephrotoxicity [14–16]. However, the protective mechanism of QC is not well understood.

In tumor cells, QC has been shown to induce cell apoptosis and exert an anti-tumor effect [36–38]. However, Aalinkeel and colleagues [39] revealed that QC selectively induced cell apoptosis in prostate cancer cells while exerting no effect on normal prostate epithelial cells. Similar to the above results, Jeong *et al.* [40] reported that relatively low doses of QC (1–10 μM) administered to breast cancer cells induce cell cycle arrest at the G0/G1 phase while the proliferation of normal breast epithelium was not affected by 10 μM QC, which suggests that QC has a cancer-specific anti-proliferative effect. Moreover, researchers found that in normal cells, QC inhibited apoptosis induced by various stimuli, such as hydrogen peroxide, glucose oxidase, and oxidized low-density lipoprotein [41–43]. The complicated effects of QC prompted us to further explore the effect of QC on ADMA-induced apoptosis. We carried out experiments and found that QC inhibited ADMA-induced apoptosis. This result is consistent with previous results in normal cells. Therefore, in normal injured cells, QC may exert a protective effect.

Because of the complexity of QC on apoptosis, the effects of QC on ER stress were also inconsistent. QC induced ER stress in cancer cells [44], while it inhibited ER stress when normal cells encountered ischemia/reperfusion injury [45]. Based on the previous observation of QC inhibiting ADMA-induced apoptosis, we speculated that QC may inhibit ADMA-induced ER stress. Indeed, we found that QC interfered with the PERK-CHOP- and IRE1-JNK-mediated apoptotic pathway. Overexpression of CHOP canceled the protective effect of QC on ADMA-induced apoptosis. We concluded that inhibition of the ER stress was an important mechanism of QC during ADMA-induced cell apoptosis.

Wiseman *et al.* [46] reported that QC activates IRE1 through a site distinct from the nucleotide-binding site. We also notice that PERK phosphorylation and IRE1 expression was higher than that of control cells when GEnCs were treated with QC alone. To explore the reasons why QC treatment induced ER stress, we examined the proliferation and change in the cell cycle upon QC treatment using the tetrazolium-based colorimetric assay (MTT) and flow cytometry. No significant changes were observed (data not shown). Other studies also indicated that concentrations of 10–50 μM QC are considered to be pharmacologically safe in endothelial cells [47–49]. According to some published results, in cancer cells, QC treatment can cause apoptosis via the ER stress pathway [44]. In our experiments, GEnCs did not show any apparent apoptosis by QC treatment, but it did cause ER stress. Therefore, QC may have contrasting effects under different conditions. As such, the mechanisms and implications of QC-induced ER stress in GEnCs remain to be elucidated.

Apoptotic cells release cytokines such as TGF-β, which is a major profibrotic mediator in CKD [50]. TGF-β stimulates the synthesis of extracellular matrix molecules including fibronectin, laminin, type I collagen and type IV collagen, which contributes to the progression of renal fibrosis [51]. In this study, we also demonstrated that ADMA induced TGF-β synthesis. Increased TGF-β synthesis was mediated by CHOP and JNK. Furthermore, QC inhibited ADMA-induced TGF-β expression. Therefore, QC may play a potential role in preventing renal fibrosis. The antifibrotic effects of QC are induced by a complex mechanism that involves the inhibition of apoptotic signaling pathways, such as PERK-CHOP and IRE1-JNK, and the reduction of apoptosis-related cytokines, such as TGF-β.

## 4. Materials and Methods

### 4.1. Chemical Reagents

ADMA and QC were purchased from Sigma-Aldrich (St. Louis, MO, USA). ATF4, PERK, p-PERK, phosphorylated c-Jun and β-actin antibodies were purchased from Santa Cruz Biotechnology, Inc. (Santa Cruz, CA, USA). IRE1, c-Jun *N*-terminal kinase (JNK), p-JNK and cleaved-caspase-3 antibodies were bought from Cell Signaling Technology (Beverly, MA, USA). CHOP/GADD153 antibody was obtained from Abcam Inc. (Cambridge, MA, USA). All other reagents, except where stated, were of molecular biology grade and were purchased from Sigma-Aldrich.

### 4.2. Cell Culture

Primary GEnCs were purchased from Sciencell Inc. (Carlsbad, CA, USA). GEnCs were cultured in fibronectin-coated flasks using endothelial cell medium (ECM, Sciencell Inc., Carlsbad, CA, USA) with bullet kit additives (Sciencell Inc.) and 10% (*v*/*v*) fetal bovine serum (Sciencell Inc.). GEnCs used in the experiments were between 3 and 4 passages.

### 4.3. Apoptosis Assays

For detection of apoptotic cells, cells were washed twice in phosphate-buffered saline (PBS) and then stained with the fluorescein isothiocyanate (FITC) Annexin V Apoptosis Detection Kit (BD Biosciences, San Diego, CA, USA) according to the manufacturer’s instructions. Thereafter, cells were analyzed by fluorescence-activated cell sorting (FACS) within 1 h on a FACS Calibur with Cell Quest software (Beckman Coulter, Fullerton, CA, USA). Cells that were annexin V-FITC- and propidium iodide (PI)-negative were viable. Early apoptotic cells stained positive for annexin V-FITC only. Cells positive for both annexin V-FITC and PI were designated as dead cells, either as a result of late apoptosis or necrosis. The percentages of annexin V-positive cells within the PI-negative population were then calculated after flow cytometric analysis.

### 4.4. Western Blot Analysis

Cells were washed twice with ice-cold PBS and harvested in RIPA buffer (50 mM Tris-HCl, pH 7.4, 150 mM NaCl, 0.25 mM EDTA, pH 8.0, 1% (*w*/*v*) deoxycholic acid, 1% (*v*/*v*) Triton X-100, 5 mM NaF, 1 mM sodium orthovanadate) containing protease inhibitors. Protein samples (20 μg) were separated on a 10% (*w*/*v*) SDS polyacrylamide and transferred to nitrocellulose membrane (Amersham International plc., Cardiff, UK) electrophoretically. Membranes were blocked with 5% (*w*/*v*) nonfat milk in tris-buffered saline (TBS) containing 0.1% (*v*/*v*) Tween 20 (TBST) for 2.5 h, followed by an overnight incubation with primary antibodies at 4 °C. The membranes were hybridized with horseradish peroxidase-conjugated secondary antibodies for 1 h, processed using the enhanced chemiluminescence (ECL) kit (Millipore Co., Billerica, MA, USA) and then exposed to Kodak X-OMAT film (Eastman Kodak Inc., New York, NY, USA). The primary antibodies used in this study were as follows: anti-CHOP, anti-ATF4, anti-p-PERK, anti-PERK or anti-β-actin.

### 4.5. Estimation of mRNA Levels

Total RNA was isolated from GEnC using Trizol (Invitrogen Life Technologies, Carlsbad, CA, USA) and reverse transcribed using the Reverse Transcription System according to the manufacturer’s instructions (Promega Corporation, Madison, WI, USA). Quantitative real-time reverse transcription-PCR (RT-PCR) was carried out using a 7300 Real-time PCR System (Applied Biosystems, Foster City, CA, USA) with Power SYBR Green PCR Master Mix (Applied Biosystems) according to the manufacturer’s instructions to quantify the expression levels of ATF4 and CHOP mRNA normalized to glyceraldehyde-3-phosphate dehydrogenase (GAPDH). All reactions were performed in triplicate with samples derived from three independent experiments. The following sequences of the forward and reverse primer pairs were used: CHOP forward primer 5′-ACC ACT CTT GAC CCT GCT TCT-3′ and reverse primer 5′-GCC ACT TTC CTT TCA TTC TCC-3′; ATF4 forward primer 5′-CCC CTT CAC CTT CTT ACA ACC-3′ and reverse primer 5′-GGG CTC ATA CAG ATG CCA CTA-3′; GAPDH forward primer 5′-AGA AGG CTG GGG CTC ATT TG-3′ and reverse primer 5′-AGG GGC CAT CCA CAG TCT TC-3′.

### 4.6. Transfection of Silencing RNA (siRNA)

Predesigned siRNAs against human CHOP (siCHOP, Invitrogen, Carlsbad, CA, USA, cat. no. 4392420) and control scrambled siRNA (siCont, Invitrogen, cat. no. 4390843) were purchased from Invitrogen Inc. The siRNA was transfected into GEnC using Lipofectamine RNAiMAX Reagent (Invitrogen) according to the manufacturer’s instructions. Briefly, cells were plated onto 6-well plates at 1.0 × 10^5^ cells in ECM without antibiotics and grown to 30%–50% confluence before transfection. The siRNAs were diluted to give a final concentration of 15 nM in Opti-MEM I (Invitrogen). Next, Lipofectamine RNAiMAX Reagent (Invitrogen) was added and the mixture was incubated at room temperature for 20 min. The cells were incubated with RNAi duplex-Lipofectamine RNAiMAX complexes in serum-free conditions for 6 h at 37 °C. The media was replaced, and cells were incubated for an additional 24 h before beginning the experiment.

### 4.7. Plasmid Preparation

For transfection of the plasmid expression vector encoding human GADD153, the DNA sequence containing the GADD153 open reading frame (ORF) flanked by HindIII/XhoI restriction sites was PCR amplified from HeLa cells. Primers were designed to introduce the Kozak sequence to increase translation (5′-AGG GAG ACC CAA GCT TGC CAC CAT GGA TAT GGA GCT TGT TCC AGC CAC-3′ and 5′-TAG ATG CAT GCT CGA GTC ACT TAT CGT CGT CAT CCT TGT AAT CTG CTT GGT GCA GAT TCA CCA-3′). The resulting fragment was inserted into HindIII-XhoI precut pcDNA3.1-FLAG (Invitrogen) to generate pcDNA3.1-GADD153-FLAG. The desired sequence was confirmed by direct DNA sequencing. The transfections were carried out by Lipofectamine LTX and PLUS Reagents (Invitrogen) according to the manufacturer’s protocol.

### 4.8. Enzyme-Linked Immunosorbent Assay (ELISA)

TGF-β1 levels were measured by ELISA using commercial assay Kits (R&D Systems, Minneapolis, MN, USA) according to the manufacturer’s instructions. The lower threshold of sensitivity of the TGF-β1 assays ranged from 1.7 to 15.4 pg/mL. The TGF-β1 concentration in the supernatants was calculated by normalizing standard twice-diluted series. Each individual sample was determined in triplicate.

### 4.9. Statistical Analysis

Data were expressed as the mean ± S.E. Comparisons between experimental groups were made by one-way ANOVA. The differences in the mean values were considered significant at *p* < 0.05.

## 5. Conclusions

In summary, the present data reveal mechanisms involved in the anti-apoptotic potential of QC on ADMA-induced GEnC apoptosis. To our knowledge, this is the first report to demonstrate that ADMA-induced GEnC apoptosis depends on the PERK and IRE1 ER stress apoptotic pathway, and that QC inhibits ADMA-induced GEnC apoptosis by targeting PERK-CHOP and IRE1-JNK.

## Supplementary Information
