# Supplementary material for: The Inhibitory Effect of Quercetin on Asymmetric Dimethylarginine-Induced Apoptosis Is Mediated by the Endoplasmic Reticulum Stress Pathway in Glomerular Endothelial Cells
Source: Int J Mol Sci. 2014 Jan 2;15(1):484–503. doi: 10.3390/ijms15010484 (PMC3907821; doi:10.3390/ijms15010484)
Supplement: Supplementary file 1 [file ijms-15-00484-s002.pdf]

Supplementary Information

Figure S1. CHOP expression was observed after transfection with siCHOP. Cells were transfected with control scrambled siRNA (siCont) or siCHOP, then incubated with or without 100 μM ADMA for 24 h. Extracts were prepared in RIPA lysis buffer and were subjected to western blotting.

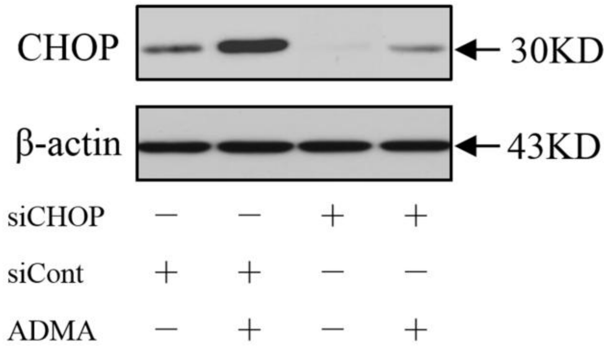

Figure S2. CHOP expression was observed after transfection with pcDNA3.1-GADD153. Cells were transfected with 2.5 μg empty vector or pcDNA3.1-GADD153. Cells were collected and subjected to western blotting.

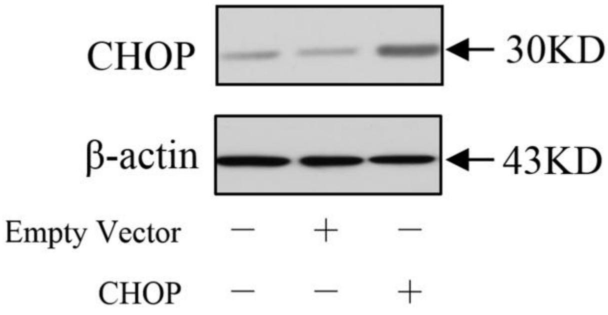

© 2014 by the authors; licensee MDPI, Basel, Switzerland. This article is an open access article distributed under the terms and conditions of the Creative Commons Attribution license (http://creativecommons.org/licenses/by/3.0/).
